# Identification and validation of a prognostic risk-scoring model for AML based on m^7^G-associated gene clustering

**DOI:** 10.3389/fonc.2023.1301236

**Published:** 2024-01-11

**Authors:** Chiyi Zhang, Ruiting Wen, Guocai Wu, Guangru Li, Xiaoqing Wu, Yunmiao Guo, Zhigang Yang

**Affiliations:** ^1^ Department of Hematology, Central People’s Hospital of Zhanjiang, Zhanjiang, China; ^2^ Zhanjiang Key Laboratory of Leukemia Pathogenesis and Targeted Therapy Research, Zhanjiang, China; ^3^ Zhanjiang Institute of Clinical Medicine, Central People’s Hospital of Zhanjiang, Zhanjiang, China

**Keywords:** acute myeloid leukemia, prognostic risk-score model, immune infiltration, m7G, prognostic biomarkers

## Abstract

**Background:**

Acute myeloid leukemia (AML) patients still suffer from poor 5-year survival and relapse after remission. A better prognostic assessment tool is urgently needed. New evidence demonstrates that 7-methylguanosine (m7G) methylation modifications play an important role in AML, however, the exact role of m7G-related genes in the prognosis of AML remains unclear.

**Methods:**

The study obtained AML expression profiles and clinical information from TCGA, GEO, and TARGET databases. Using the patient data from the TCGA cohort as the training set. Consensus clustering was performed based on 29 m7G-related genes. Survival analysis was performed by KM curves. The subgroup characteristic gene sets were screened using WGCNA. And tumor immune infiltration correlation analysis was performed by ssGSEA.

**Results:**

The patients were classified into 3 groups based on m7G-related genebased cluster analysis, and the differential genes were screened by differential analysis and WGCNA. After LASSO regression analysis, 6 characteristic genes (including CBR1, CCDC102A, LGALS1, RD3L, SLC29A2, and TWIST1) were screened, and a prognostic risk-score model was constructed. The survival rate of low-risk patients was significantly higher than that of high-risk patients (p < 0.0001). The area under the curve values at 1, 3, and 5 years in the training set were 0.871, 0.874, and 0.951, respectively, indicating that this predictive model has an excellent predictive effect. In addition, after univariate and multivariate Cox regression screening, histograms were constructed with clinical characteristics and prognostic risk score models to better predict individual survival. Further analysis showed that the prognostic risk score model was associated with immune cell infiltration.

**Conclusion:**

These findings suggest that the scoring model and essential risk genes could provide potential prognostic biomarkers for patients with acute myeloid leukemia.

## 1 Introduction

Acute myeloid leukemia (AML) is a heterogeneous disease at the genetic level caused by the accumulation of mutations in hematopoietic stem and progenitor cells (1). Although the remission rate of AML has been significantly improved due to the development of therapeutic modalities such as combination chemotherapy, hematopoietic stem cell transplantation, and targeted therapy, the 5-year survival rate of patients with AML remains poor and relapse after remission has become a major cause of poor prognosis (2). In recent years, studies for prognostic assessment of AML have been carried out and either genetic or cytogenetic abnormalities as a more comprehensive risk stratification system have provided effective therapeutic guidance for the clinical management of AML patients (3). Meanwhile, structural genomic alterations and targeted sequence-based screening have also provided important tools for clinical diagnosis and risk stratification of AML, enabling favorable treatment options for low-risk and high-risk patients. However, appropriate treatment option for patients in the intermediate-risk group remains unsatisfied (4). Therefore, a more comprehensive prognostic risk scoring model is urgently needed for accurate treatment option and precise prognosis in patients with AML.

Accumulated evidence suggests that RNA modifications play a critical role in various malignancies. More than 170 types of RNA modifications have been documented and are not limited to mRNA, rRNA, and tRNA (5–7). N7-methylguanosine (m^7^G) modifications are widely present within the RNA of living organisms (8–14). The understanding of the regulators of m^7^G modifications is still in the preliminary stage. Identified m^7^G regulators in mammals include RNMT/RAM, METTL1/WDR4 and WBSCR22/TRMT112 (9). As an universal RNA modification, m^7^G modification is not only required for eukaryotic mRNA translation but is also present within rRNA and tRNA of all species (8–14). It has been shown that Methyltransferase-like 1 (METTL1), a regulator of m^7^G in mammals, binds to the cofactor WD repeating domain 4 (WDR4) and installs m^7^G modifications in tRNAs, miRNAs and mRNAs (9). rRNA guanine-7 methyltransferase (RNMT) is also involved in the regulation of m^7^G modification at the 5’ cap of mRNA upon binding to the cofactor RNMT activates microproteinsl (RAM) (15). 18S rRNA m7G modification in humans is installed by a complex formed by Willimams-Beuren syndrome chromosome 22 region (WBSCR22) and TRM112-like protein(TRMT112) (16). METTL1 and WDR4 are significantly overexpressed in AML samples and knockdown of METTL1 effectively inhibits the growth of leukemic stem cells (17). A recent transcriptome-wide study of differential m^7^G methylationome profiling showed that drug-resistant AML cells had significantly different levels of m^7^G mRNA modification from AML cells and were significantly enriched in drug resistance-associated mRNA (18). These findings suggest that m^7^G methylation modifications have potential predictive value for the prognosis of AML.

In the present study, based on the gene expression data of AML samples from The Cancer Genome Atlas (TCGA) and Gene Expression Omnibus (GEO) databases, a cluster analysis was performed with m^7^G-related genes to interrogate the genetic characteristics of AML patients in different subgroups. Moreover, by analyzing the characteristic genes of each subgroup, a new prognostic model was constructed, and the relationship between the prognostic model and tumor immune microenvironment (TIM) was further investigated.

## 2 Materials and methods

### 2.1 Data download and pre-processing

TCGA and GEO data were downloaded and pre-processed using R software, and RNA sequencing (RNAseq) data were downloaded using the “TCGAbiolinks” package on May 3rd, 2022. During the data download phase, we used the “GDCquery” function in the “TCGAbiolinks2.25.2” package and selected the data type “HTSeq-count,”; used the “GDCdownload” function to download the results, and the “GDCprepare” function to convert the results into R language processable SE (SummarizedExperiment) files. The “GDCquery_clinic” function in the “TCGAbiolinks 2.25.2” package of R software is used to download the relevant clinical information, and the download time is also on May 3rd, 2022. The “TCGAanalyze_Preprocessing” function in the “TCGAbiolinks 2.25.2” was used for data preprocessing. Removing outliers from data using spearman correlation coefficient. The “SummarizedExperiment” package was then used to annotate the expression matrix genes.

Genomic, transcriptomic, and matched clinical data for patients with metastatic uroepithelial carcinoma treated with anti-PD-L1 agents are available under a Creative Commons 3.0 license and can be downloaded from http://research-pub.gene.com/IMvigor210CoreBiologies. The corresponding dataset(miniml format) was downloaded from the GEO website, from which the corresponding expression matrix and clinical information were extracted.

### 2.2 Consensus clustering and identification of related gene sets

#### 2.2.1 Gene screening

We identified m^7^G-related genes from the published literature. We identified gene sets named “m^7^G(5`)pppN diphosphatase activity”, “RNA 7-methylguanosine cap binding,” and “RNA cap binding” from the Molecular Characterization Database (MSigDB, https://). binding” and “RNA cap binding” (MSigDB, https://www.gsea-msigdb.org/gsea/msigdb/search.jsp).

#### 2.2.2 Consensus clustering

AML patients from the TCGA database were divided into groups based on the expression of 29 m^7^G-related genes using the “ConsensusClusterPlus” R package. Then the “survival” R package was used for Kaplan-Meier (KM) complete survival curve analysis was then performed between the different clusters using the “survival” R package. Principal component analysis (PCA) was applied to evaluate sample clustering.

#### 2.2.3 Collection of subtype trait genes

Weighted gene co-expression network analysis (WGCNA) was performed by R software. The three clusters obtained by consensus clustering were used as the basis to run WCGNA to obtain the set of three subgroup correlation feature genes, firstly to obtain the adjacency matrix by weighted correlation coefficients. Next, the adjacency matrix was converted into a topological Topological overlap matrix (TOM). Then, hierarchical clustering was performed to identify the modules and to calculate the characteristic genes. Finally, correlations between different isoforms and each module were evaluated by Pearson correlation analysis, and each isoform-related module was identified. The genes in these modules were considered subtype-associated module genes. KEGG pathway enrichment analysis was performed for each subtype-associated signature gene using the “clusterProfiler” package in R software.

### 2.3 Construction and validation of the prognostic model

#### 2.3.1 Construction of a prognostic risk score model based on m^7^G-related gene correlation clustering

To select genes associated with prognosis (*p* < 0.005), we performed a one-way Cox regression analysis on these co-expressed genes. Then 80% of the samples were randomly selected for lasso regression analysis and repeated 1000 times, finally retaining the genes with a frequency of 500 occurrences or more. The lasso regression was then applied to remove redundant prognostic genes, and finally, the characteristic genes were retained to develop a prognostic assessment model. Survival” and “survminer” R packages were used to compare the survival differences between the high-risk and low-risk groups, and the “time-ROC” R package was used to The 1-, 3- and 5-year ROC curves were analyzed to verify the model prediction accuracy. The AML data from GEO (GSE71014) and TARGET database (TARGET-AML) were used as test data sets for risk score calculation, risk subgroup assessment, survival analysis, and ROC curve plotting in the same way.

#### 2.3.2 Prognostic analysis of the prognostic risk score model

Further clinical data such as age, gender, race, category, and risk score were extracted from the TCGA cohort, and univariate and multivariate Cox regression analyses were performed to identify independent prognostic factors.

#### 2.3.3 Creation of column line diagram

Column plots were constructed with the R software packages “rm” and “regplot” to visualize the relationship between the variables and the prognostic model. Calibration curves at 1, 3, and 5 years were applied to differentiate and predict the values of the line graphs. To better illustrate the role of risk scores in developing AML, we analyzed the relationship between risk scores and different clinical characteristics.

#### 2.3.4 Correlation of immune infiltration prognostic models

Comparison of immune cell differences between high and low risk groups using the ssGSVA algorithm. The ssGSVA algorithm assessed the content of different immune cells between high and low-risk groups. The anti-cancer immune response can be conceptualized as a series of stepwise events known as the cancer-immune cycle. We obtained correlations between prognostic scores and anti-cancer immune status in a seven-step cancer-immune cycle through analysis at the TIP online website (http://biocc.hrbmu.edu.cn/TIP/index.jsp), including cancer antigen release (step 1), cancer antigen presentation (step 2), initiation and activation (step 3), immune cell to tumor transport (step 4), infiltration of immune cells into the tumor (step 5), recognition of cancer cells by T cells (step 6), and killing of cancer cells (step 7).

#### 2.3.5 Mutation distribution of the prognostic model

AML mutation data were obtained from the TCGA database, and the R package “maftools” was used to visualize somatic mutations between the high-risk and low-risk groups.

#### 2.3.6 Prognostic model for chemotherapy response prediction

Due to the lack of drug data in the TCGA-LAML dataset, we used the immunotherapy dataset for bladder cancer (IMvigor210 cohort) to predict chemotherapy response for our prognostic model. The efficiency of the prognostic model was validated using the risk score distribution of patients with different drug response groups.

### 2.4 Quantitative PCR assay

Total RNA was extracted from peripheral blood mononuclear cells (PBMCs) with Trizol reagent (TaKaRa, Tokyo, Japan), according to the manufacturer’s instructions. RNA concentration was measured *via* Infinite 200PRO (Science & Technology, Männedorf, Swiss).Subsequently, cDNA synthesis and quantitative PCR (qPCR) were performed by a reverse transcriptase kit (Vazyme) and an SYBR Premix Ex Taq™ II kit (Vazyme), respectively, each according to the manufacturer’s instructions. All of the primers used were synthesized by SangonBiotech (China, Guang zhou) and they are listed in **Table 1**. Relative expression levels of CBR1, CCDC102A, LGALS1, SLC29A2, RD3L and TWIST1 were quantified according to the 2^-ΔΔCt^.

**Table 1:** Primers used for qPCR assays.

Primer name	Sequence(5’ to 3’)
CBR1-F	AGCTGGACATCGACGATCTGCA
CBR1-R	TATGAAAGGGTGTGGGATCAGCA
SLC29A2-F	GGATCTTGACCTGGAGAAGGAG
SLC29A2-R	GTGAAGACCAACACAAGGCACAG
LGALS1-F	AGCAGCGGGAGGCTGTCTTTC
LGALS1-R	ATCCATCTGGCAGCTTGACGGT
RD3L-F	AAAATGGCACCTAAAGGAGCG
RD3L-R	TCTGGTAGTTTCTGAGCCAGTT
TWIST1-F	GCCAGGTACATCGACTTCCTCT
TWIST1-R	TCCATCCTCCAGACCGAGAAGG
CCDC102A-F	AGTCCCAGAAGGTGCTGCTCAA
CCDC102A-R	GAGCATCTCCTGCTTGGTCTTG

### 2.5 Statistical analysis

All experiments were performed in at least three biological replicates, and each biological replicate contained three technical replicates, and the experimental data were analyzed in GraphPad Prism 9. The Data were presented as mean ± standard deviation (SD). Statistical details were calculated by Student’s t test (for two groups, paired comparison for clinical patient data). *P* values less than 0.05 were considered statistically significant.

## 3 Results

### 3.1 Consensus clustering based on m^7^G-related genes

After searching the previous studies, 29 m^7^G-related genes were selected, including *AGO2*, *CYFIP1*, *DCP2*, *DCPS*, *EIF3D*, *EIF4A1*, *EIF4E*, *EIF4E1B*, *EIF4E2*, *EIF4E3*, *EIF4G3*, *GEMIN5*, *IFIT5*, *LARP1*, *LSM1*, *METTL1*, *NCBP1*, *NCBP2*, *NCBP2L*, *NCBP3*, *NSUN2*, *NUDT10*, *NUDT11*, *NUDT16*, *NUDT3*, *NUDT4*, *NUDT4B*, *SNUPN*,and *WDR4*. The distribution of these genes on the chromosomes is shown in **Supplementary Figure 1A**. Somatic mutation analysis showed that most genes were not mutated in AML samples (**Supplementary Figure 1B**).

**Figure 1:**
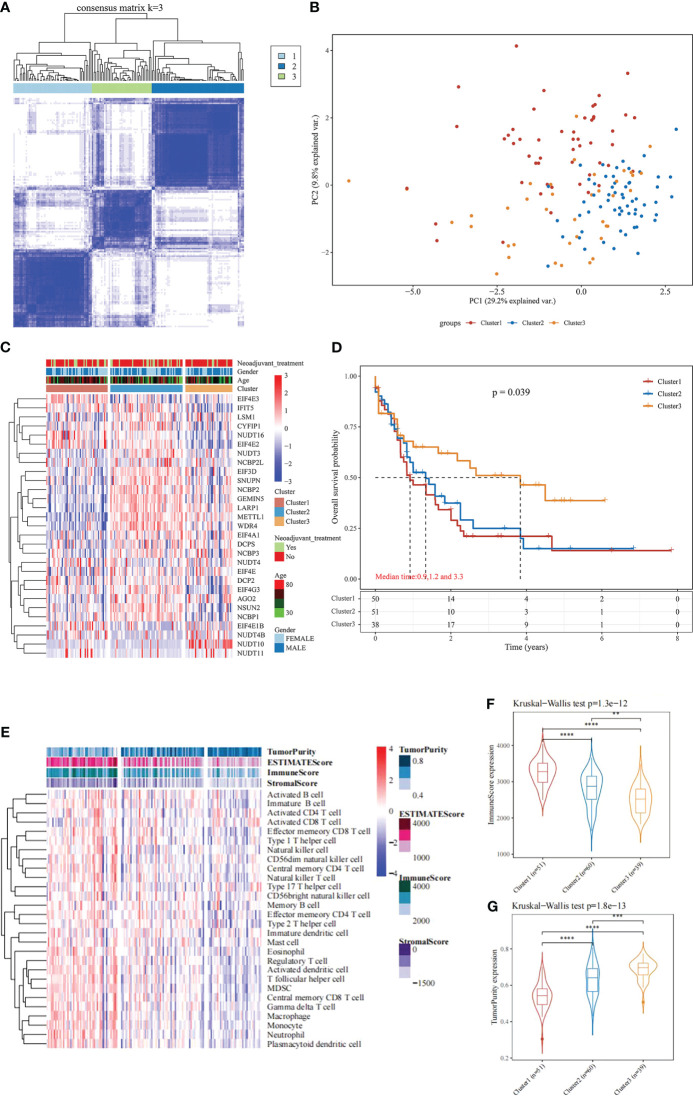
Consensus Clustering and survival analysis. **(A)** Consensus clustering heat map; **(B)** PCA plot; **(C)** m7G gene expression heat map; **(D)** Kaplan-Meier survival analysis between different subgroups; **(E)** Heat map and differences in immune infiltration and tumor purity between the three groups; **(F)** Comparison of immunescore between the three groups; **(G)** Comparison of tumorpurity between the three groups. **P < 0.01, ***P < 0.001, and ****P < 0.0001.

Based on the expression similarity of the 29 m^7^G-related genes, the TCGA data were clustered by applying consensus clustering. The 150 AML samples could be well divided into 3 clusters (**Figures 1A, B**), namely cluster 1 (n = 51), cluster 2 (n = 60) and cluster 3 (n = 39). The expression of m^7^G-related genes was significantly higher in cluster 2 than in the other two groups (**Figure 1C**). Moreover, further application of Kaplan-Meier survival analysis showed that cluster 1 and cluster 2 had worse prognosis as compared with cluster 3 in terms of overall survival (OS) (**Figure 1D**).

To further understand the underlying reasons for the differences in survival among the 3 clusters, the immunity, stromal score and tumor purity of the samples in each cluster were assessed using the “estimate” package. The differences in the immune microenvironment among the 3 immune clusters were explored using the ssGSEA algorithm. The results of this analysis showed that cluster 3 had fewer infiltrating immune cells and lower immune scores than cluster 1 and cluster 2 (**Figures 1E, F**), and tumor purity was significantly higher in cluster 3 (**Figure 1G**, rank sum test *P* < 0.001). These findings suggest that patients with less immune infiltration and lower immune scores have better survival than those with more immune infiltration and higher immune scores in the consensus clustering based on the m^7^G correlation.

### 3.2 Prognostic assessment model based on the construction of immune infiltration differences

**Figure 2:**
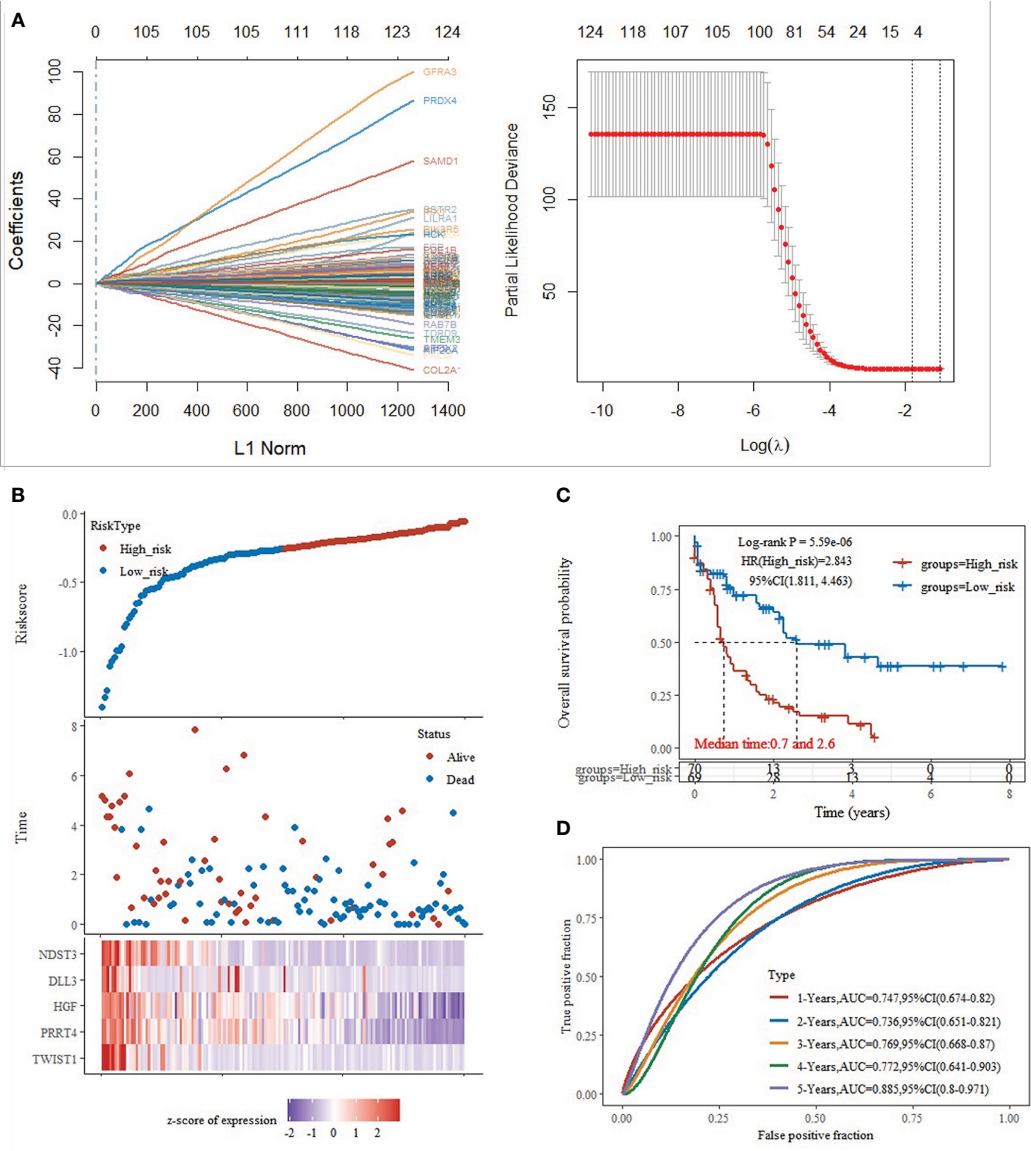
Training set model construction. **(A)** Lasso coefficient diagram,Model equation = -0.1203*exp^TWIST1^-0.0326*exp^PRRT4^ -0.0202*exp^HGF^ -0.0001*exp^DLL3^ -0.0827*exp^NDST3^; **(B)** Risk score, survival time, and survival analysis; **(C)** KM survival curve distribution; **(D)** ROC curve with AUC.

Based on the significant differences in immune infiltration and prognosis between various clusters, we defined cluster 3 as lacking immune infiltration (IL type) and cluster 1/cluster 2 as the subtype rich in immune infiltration (IR type). A detailed analysis of mRNA expression profiles of AML patients from both types was performed to reveal the potential mechanisms underlying the different prognosis between IL and IR subtypes. After gene expression analysis, 265 differential expression genes between IL and IR subtypes were identified with FDR < 0.05 and |log FC| > 2, of which 131 were upregulated in IR type, and 134 were downregulated in IR type (**Figure 2A**). One-way Cox regression analysis of the differential genes yielded 129 genes significantly associated with prognosis, which were further screened by lasso cox regression analysis to finally retain the prognostic assessment model based on five genes (**Figures 2A, B**) and validate the model with external data (GEO, TARGET data) (**Supplementary Figure 2**). The model showed good prediction accuracy in the training set [5-year, the area under the curve (AUC)= 0.885, 95% CI (0.800-0.971)] (**Figures 2C, D**). However, it failed to show the expected results in both 2 training sets [GEO: 5-year, AUC= 0.539, 95% CI (0.382-0.697); TARGET: 5-year, AUC= 0.553, 95% CI (0.467-0.639)] (**Supplementary Figure 2**). It indicates that a valid prognostic assessment model could not be obtained using immune infiltration and differential tumor purity genes as the basis for constructing the prognostic model.

### 3.3 Identification of feature gene sets

**Figure 3:**
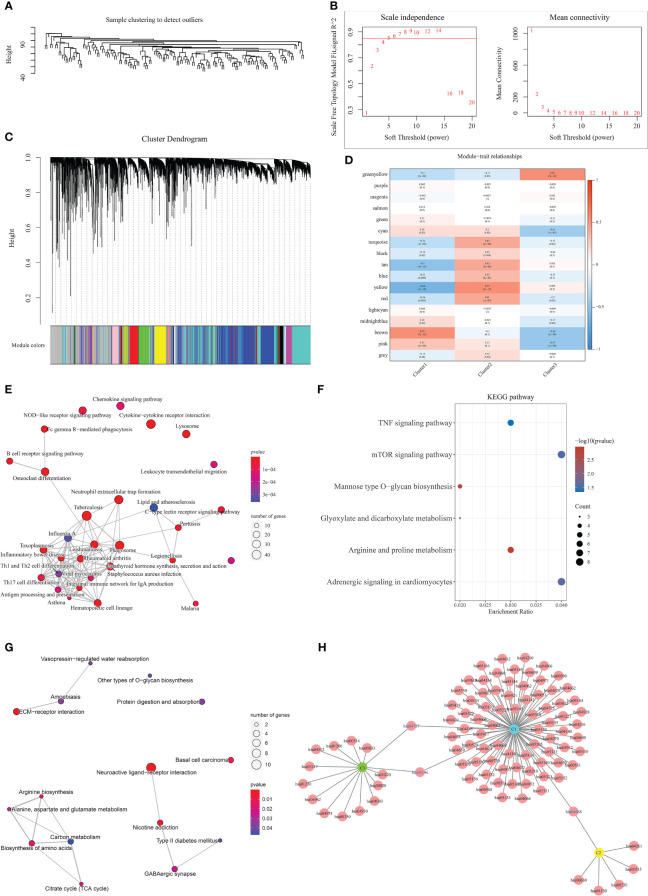
WGCNA identifies the set of feature genes. **(A)** Sample clustering analysis; **(B)** Analysis of network topology for various soft-thresholding powers; **(C)** Gene dendrogram and module colors; **(D)** Correlation results between the 17 modules and individual clinical phenotypes; **(E)** KEGG pathway analysis of brown module; **(F)** KEGG pathway analysis of yellow module; **(G)** KEGG pathway analysis of green-yellow module; **(H)** network diagram analysis of the three modules.

To further explore the differences between the above 3 clusters and to establish a prognostic assessment model with better prognostic assessment, the WGCNA algorithm were used to mine the set of co-expressed coding genes of each cluster, both the characteristic genes of each cluster. The samples were first clustered using hierarchical clustering (**Figure 3A**); further, the distance between each gene was calculated using Pearson’s correlation coefficient, and the R package WGCNA was used to construct a weighted co-expression network. Firstly, a soft threshold selection was performed to reduce the noise in the calculation of gene-gene correlations. 8 was the power with an R-squared greater than 0.85 and the first stable R-squared value, which should be selected to filter the co-expression modules (**Figure 3A**). To ensure the network is scale-free, we choose β= 8 (**Figure 3B**). In the next step, the expression matrix is converted into an adjacency matrix, which is then converted into a topology matrix. Based on the TOM, the average-linkage hierarchical clustering method were use to cluster the genes according to the criteria of a hybrid dynamic shear tree and set the minimum number of genes per gene network module 50. After using the dynamic shear method in determining the gene modules, we calculate each in turn. After using the dynamic shearing method to determine the gene modules, we calculate the eigenvector values of each module in turn and then perform cluster analysis on the modules, and merge the closer modules into new modules (height=0.25, deepSplit=3, minModuleSize=50) to obtain a total of 17 modules (**Figure 3C**), the “grey” module shows the genes that could not be aggregated to other modules. The correlation of each module with subtypes were further analyzed. The findings showed that the gene sets in “brown”, “yellow,” and “green-yellow” gene sets were significantly correlated with Cluster 1, cluster 2, and cluster 3, respectively (**Figure 3D**).

KEGG pathway analysis of the genes contained in the “brown” (1039), “yellow” (574), and “green-yellow” (147) modules were performed, respectively. KEGG pathway analysis revealed that the genes of the brown module associated with cluster 1 subtype were enriched in 78 pathways (**Figure 3E**), mainly in immune-related pathways such as phagocytic vesicles, neutrophil extracellular trap formation, and B-cell signaling pathway (**Figure 3E**). The genes of the yellow module associated with cluster 2 subtype were enriched in 6 pathways, mainly in arginine and proline metabolism, mTOR signaling pathway, TNF signaling pathway and other pathways (**Figure 3F**). Cluster 3 isoform-associated green-yellow module genes were enriched to 15 pathways, mainly in neuroactive ligand-receptor interaction, ECM-receptor interactions, citric acid cycle and other pathways (**Figure 3G**). Further analysis showed that the 3 subtypes were jointly enriched to only 3 pathways by network diagram analysis (**Figure 3H**), indicating that the 3 subtypes presented significant functional differences in the related gene modules. Therefore, the differences in the expression of these module genes may be the reason for the survival differences among the 3 subtypes.

### 3.4 Identification and validation of prognostic models

**Figure 4:**
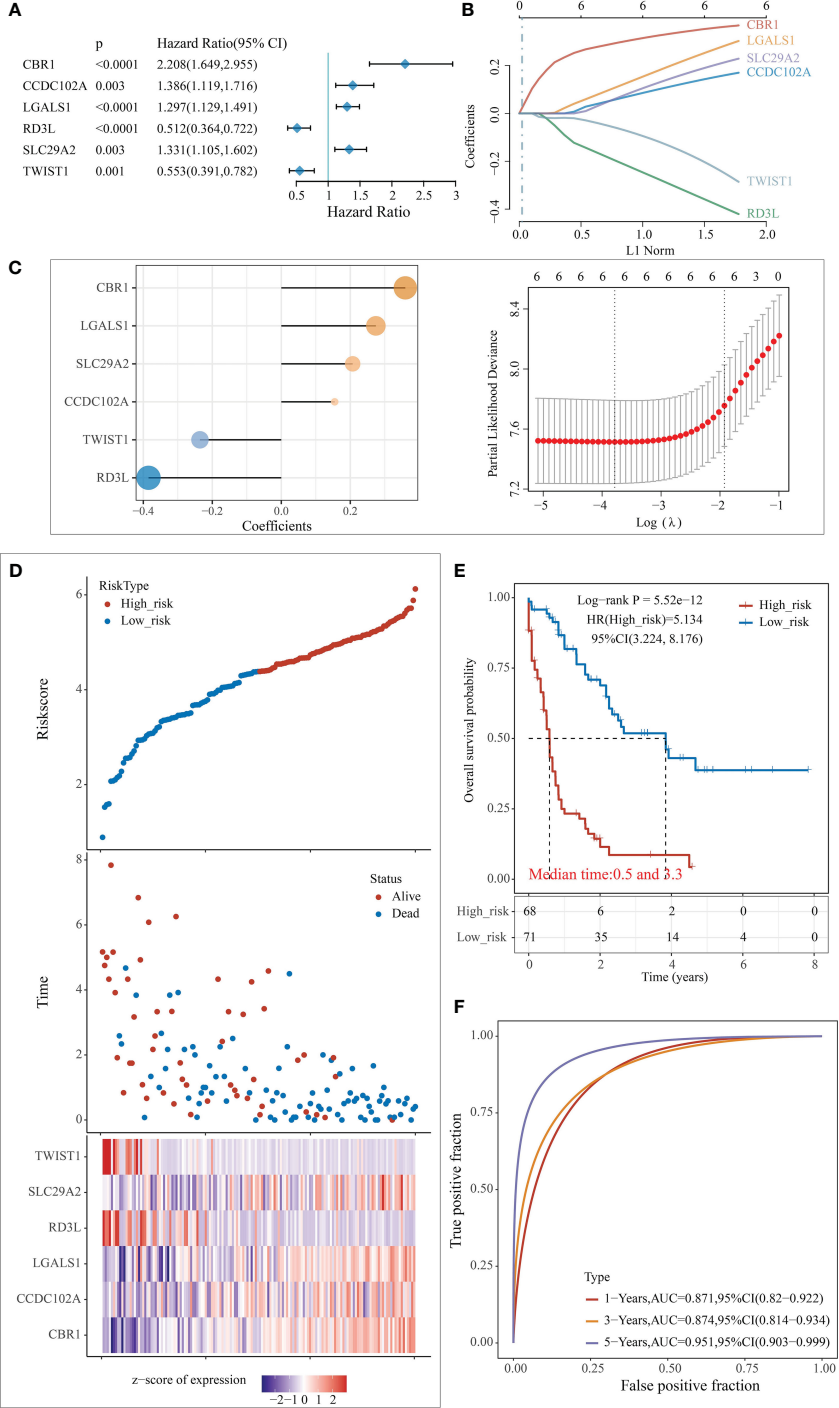
Construction of the predictive risk model. **(A)** Forest plot of 6 model genes; **(B)** Lasso variable screening process; **(C)** Lasso regression selection of 6 for prognostic genes to build a prognostic model. risk score = 0.3595*exp^CBR1 +^ 0.^1548*expCCDC1 102A^ + 0.^2741*expLGALS1^ - 0.3848*exp^RD3L^ + 0.2069* exp^SLC1 29A2^ - 0.2357^*expTWIST1^; **(D)** Risk score, survival time, and survival analysis; **(E)** KM survival curve distribution; **(F)** ROC curve with AUC.

Based on the enrichment analysis of 3 clusters of modular genes, 1754 differential genes between co-expression modules were obtained. Univariate Cox regression model analysis was performed on these genes’ expression and survival data in the TCGA training set samples. 194 differential genes that were significantly associated with prognosis were obtained (*p* < 0.005). To further screen the genes used for model building, 80% of the TCGA training set samples for lasso regression analysis were randomly selected, using tenfold cross-validation, thus performed 1000 times of lasso analysis, finally retained 6 mRNAs with frequencies > 500 as target genes (**Figure 4A**). R package glmnet was used for lasso regression analysis and finally obtained the optimal model parameters (**Figures 4B, C**).

The risk score for each sample was separately calculated according to the expression level of the samples. The risk score distribution of the samples was plotted (**Figure 4D**), from which it can be seen that samples with high-risk scores exhibit worse OS, which suggests that samples with high-risk scores have a worse prognosis. High expression of CBR1, CCDC102A, LGALS1, and SLC29A2 are associated with high risk as risk factors; high expression of RD3L and TWIST1 was associated with low risk and were protective factors.

The samples were classified into high and low risk groups based on the above median risk scores, and a significant difference between the two groups could be seen from the plotted KM curves (log rank p < 0.0001, HR = 5.134), in which 68 samples were classified as high risk and 71 as low risk (**Figure 4E**). ROC analysis for prognostic classification of risk scores were further performed using the R package timeROC, and the prognostic predictive classification efficiency at 1, 3, and 5 years was analyzed, as shown in **Figure 4F**, from which we can see that the model has a high AUC, all above 0.87.

The model was further validated in the external validation set using the same model and coefficients as in the test data set (**Supplementary Figures 3**, **4**). The KM plots based on risk scores in the validation set all divided the sample well into significantly different high and low-risk groups (GSE71014: log-rank *p* = 0.0271, HR = 2.345; TARGET-AML: log-rank *p* = 0.0478, HR = 1.63) (**Supplementary Figures 3A, B**, **4A, B**). The GSE71014 dataset shows the model has a high AUC area under the line, with 1-year and 3-year AUCs above 0.6 (**Supplementary Figure 3C**). The 1-year AUC of the model out of TARGET-AML is 0.739, while the 3 and 5-year AUCs are lower (**Supplementary Figure 4C**).

### 3.5 Independent prognostic analysis of risk scores and clinical and pathological features

**Figure 5:**
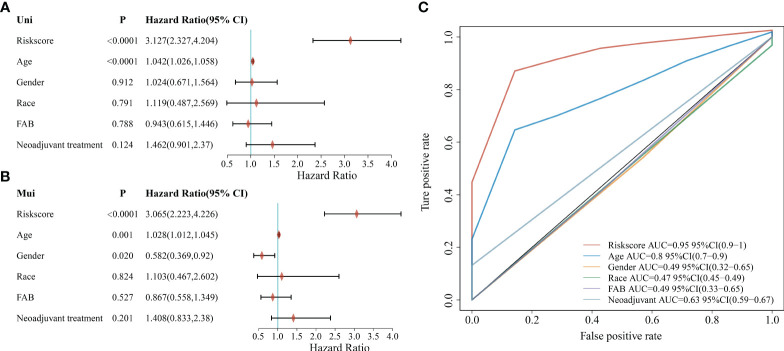
Correlation between clinicopathological characteristics, risk score, and prognostic value in the TCGA cohort. **(A)** Univariate analysis of clinicopathological factors and risk scores; **(B)** Multivariate analysis of clinicopathological factors and risk scores; **(C)** ROC curve showing the predictive effect of risk scores and clinicopathological characteristics.

To further explore the clinical value of the prognostic risk score model, univariate and multivariate Cox regression analyses were performed on the TCGA cohort. In the univariate Cox analysis, age and risk score were significantly associated with the prognosis of AML patients (**Figure 5A**). In addition, the results of multivariate Cox regression indicated that age and risk score were independent risk factors for AML patients’ prognosis (**Figure 5B**). Next, we performed a ROC analysis of these factors, and the results of the AUC values indicated that the risk score had higher accuracy in predicting OS than the risk factor of age (**Figure 5C**).

Using age, sex, race, FAB typing, and adjuvant therapy as characteristics in the TCGA dataset sample for grouped survival analysis, risk scores with six characteristic genetic markers could significantly distinguish the young group, old group, male, female, different races, FAB typing and patients without adjuvant therapy between the two groups of high and low risk (*p* < 0.05, **Supplementary Figure 5**), which also further indicates that the model has good predictive power in patients with different clinical characteristics. In addition, the correlation between risk scores, clinical characteristics, and pathological staging were further analyzed. The results showed that the risk score was significantly correlated with the patient’s age and FAB typing (*p* < 0.05, **Supplementary Figure 6**).

### 3.6 Creation of predictive column line graphs for AML patients

**Figure 6:**
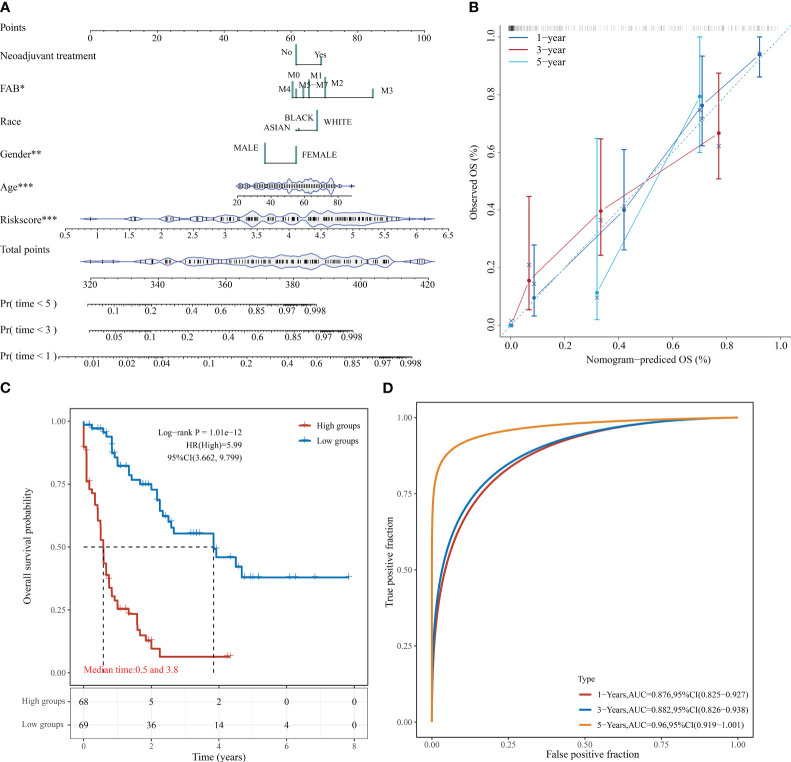
Column line diagram of TCGA-LAML. **(A)** Columnar line graph predicting 1-, 3-, and 5-year OS in AML patients; **(B)** Calibration curve of the columnar line graph; **(C)** KM curve of the columnar line graph model; **(D)** AUC curve of the columnar line graph model at 1, 3, and 5 years. *P < 0.05, **P < 0.01, and ***P < 0.001.

Based on the regression analysis described above, a column line plot we developed that provides clinicians with a quantitative approach to prediction. A score is obtained for each prognostic parameter for each patient, and the corresponding survival prediction can be retrieved in the table using the resulting total score (**Figure 6A**). In addition, the calibration plot curve fragmentation plot also shows that the column line plot has a better predictive function than the ideal model (**Figure 6B**). The samples were further divided into high and low-risk groups based on the column line plot model scores with median values and plotted KM curves, from which it can be seen that there was a significant difference in OS between high and low-risk groups (log-rank p < 0.0001, HR = 5.99), with 68 samples classified as high risk and 69 samples as low risk (**Figure 6C**). The ROC analysis was used to validate the classification efficiency of the column line plot for prognostic prediction at 1, 3, and 5 years, from which it can be seen that the column line plot scoring model has a high area under the AUC line, with AUC above 0.876 at 1, 3, and 5 years (**Figure 6D**).

### 3.7 Correlation between risk model and cellular characteristics of immune infiltration

**Figure 7:**
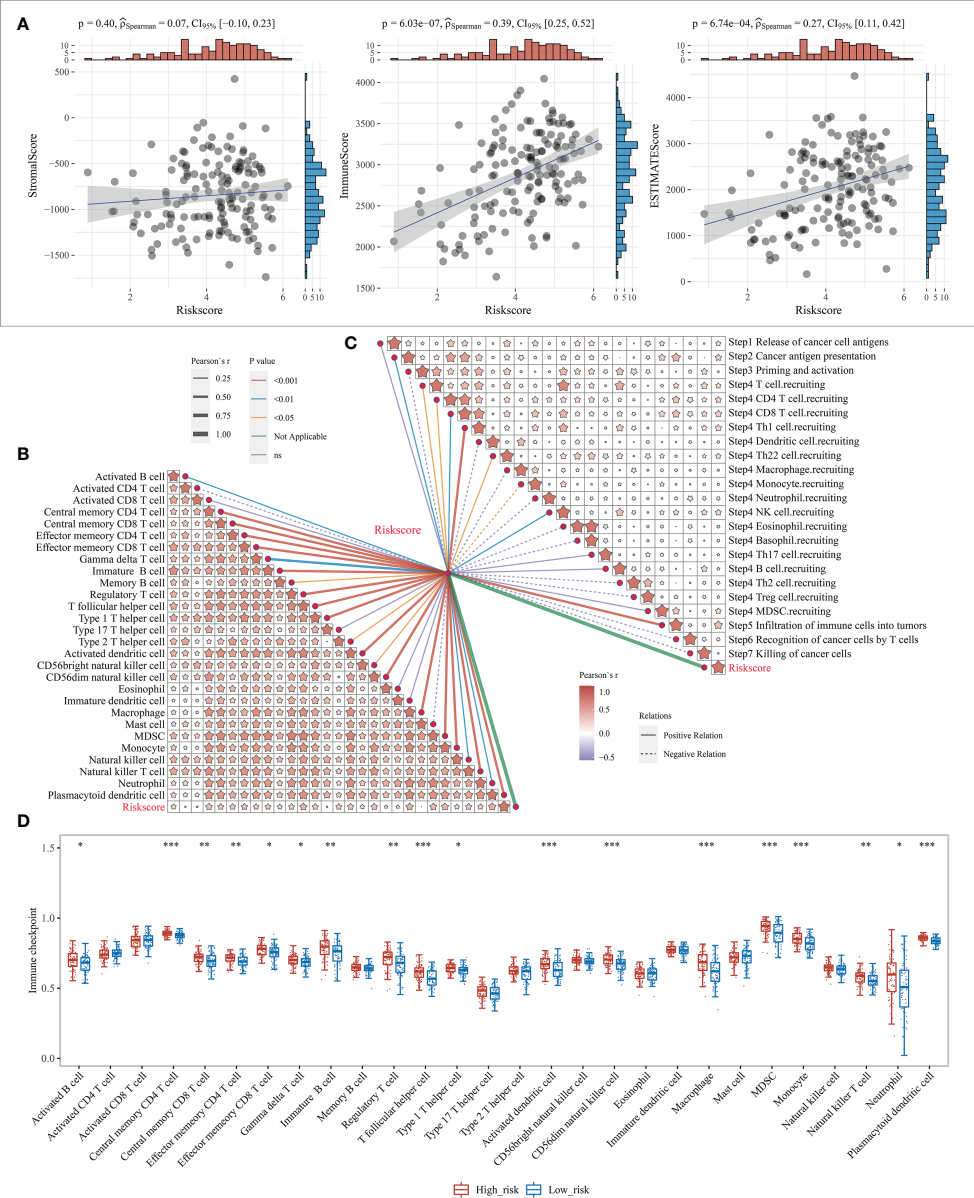
Level of immune infiltration for prognostic features. **(A)** Correlation between risk model and different immune infiltrating cell types; **(B)** correlation between risk model and immune cell infiltration scores; **(C)** Correlation between risk model and cancer immune cycle steps, where different colors of connecting lines represent different p-values, dashed and solid lines represent negative and positive correlations, respectively, and the thickness of the lines represents the size of correlation coefficients, the thicker the correlation coefficients are, the more significant the correlation coefficients are, and different colors in the same table represent the magnitude of correlation coefficients; **(D)** Correlation between risk scores and 28 types of TIL subgroups. *P < 0.05, **P < 0.01, and ***P < 0.001.

Our previous study showed that the survival differences based on m^7^G correlation clustering may correlate with the immune infiltration and tumor purity of each group. Here, the relationship between the risk score and the immune cell score was further analyzed by using the R software “estimate” package. The results showed that both the immune cell and mechanism scores showed a significant positive correlation with the risk model, with the correlation coefficients at 0.39 and 0.27, respectively (**Figure 7A**). This also indicates that there is a link between risk model expression and tumor immunity. It also further indicates the relationship between the risk model and cellular immunity.

The anti-cancer immune response can be conceptualized as a series of stepwise events known as the cancer-immune cycle, including the release of cancer cell antigens (Step1), cancer antigen presentation (Step2), initiation and activation (Step3), transport of immune cells to the tumor (Step4), infiltration of immune cells into the tumor (Step5), recognition of cancer cells by T cells (Step6), and killing of cancer cells (Step7). Here, the 7-step outcome matrix was obtained through the Tracking Tumor Immunophenotype (TIP) website analysis, and similarly analyzed the risk model correlation with the 7-step anti-cancer immune response. The results showed that Step2, Step4 T-cell recruitment, Step4 CD4 T-cell recruitment, Step4 CD8 T-cell recruitment, Step4 Th1 cell recruitment, Step4 Th22 cell recruitment, Step4 NK cell recruitment, and Step5 immune cell infiltration into the tumor showed a positive correlation with the risk model, while Step4 monocyte recruitment showed negative correlation (**Figure 7C**). ssGSEA algorithm was further used to identify 28 TIL subpopulations, including the main types associated with adaptive immunity: activated T cells, Tcm, TemCD4^+^ and CD8^+^ T cells, Tγδ cells, Th1, Th2, Th17, regulatory T cells, follicular T cells, activated B cells, immature B cells, and memory B cells; and cells associated with innate immunity types, such as macrophages, monocytes, mast cells, eosinophils, neutrophils, activated DCs, plasma cell-like and immature DCs, NK cells, NKT cells and MDSC (**Figure 7B**, **Supplementary Figure 7**). The results revealed that the risk model showed a significant positive correlation with the vast majority of the 28 TIL subpopulations (**Figure 7D**).

### 3.8 Mutation analysis of risk score models and prognostic models for immunotherapy response

**Figure 8:**
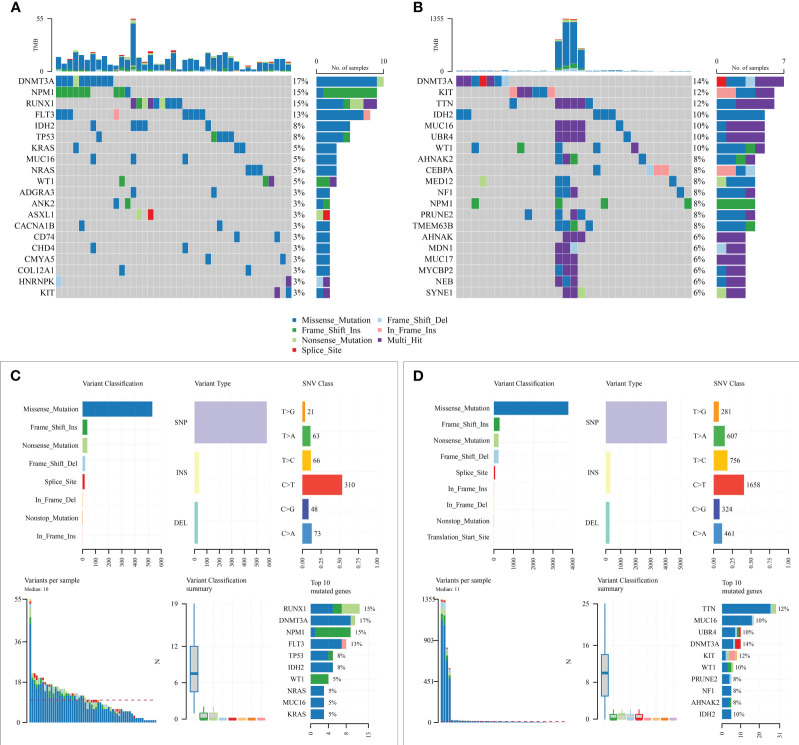
Mutation state analysis of the prognostic model. **(A, B)** Mutation status of high-risk and low-risk groups; **(C, D)** Summary of detailed mutation information of high-risk and low-risk groups.

Subsequently, mutation patterns between the two risk groups were compared. The results showed that higher samples mutated in the high-risk group than in the low-risk group and with significantly more known AML high-risk molecular mutations in the high-risk group (**Figure 8**).

Due to the lack of drug information for AML patients, the bladder cancer immunotherapy cohort (IMvigor210) was used to predict the efficacy of chemotherapy for both risk groups. The risk score was first calculated for each sample according to the model, and then the best cut-off value (3.537524) was found by the R software survminer package to divide the two high and low-risk groups (**Supplementary Figure 8A**), log-rank *p* = 0.0328; the treatment response rate was higher in the low-risk group compared to the high-risk group (26.32% vs. 21.99%, **Supplementary Figure 8B**). Unfortunately, the differences in the distribution of risk scores among the four groups of patients with different treatment responses were not observed (*p* = 0.27, **Supplementary Figure 8C**).

### 3.9 Verification of key risk genes in AML patients

Finally, six key risk genes with CBR1, CCDC102A, LGALS1, SLC29A2, RD3L and TWIST1 were examined for expression levels using qPCR in 10 AML patients and 10 healthy donors. There was no significant difference in the expression levels of CBR1, CCDC102A, LGALS1, SLC29A2, RD3L and TWIST1 genes between AML patients and healthy donors (**Supplementary Figure 9**). The reason for the differences between these validation results and the above results is related to the small sample size included.

## 4 Discussion

AML is the most common acute leukemia in adults, with a high mortality rate and poor prognosis. Chemotherapy remains the main option for most AML patients. Recent studies have shown that m^7^G-related regulators are highly expressed in AML and can affect the proliferation of leukemic stem cells; also, m^7^G mRNA modification levels are significantly elevated in drug-resistant AML cell lines (17, 18). However, there is still a lack of studies of specific mechanisms by which m^7^G affects AML development and treatment, and its relevance to AML prognosis still needs further investigation.

In this study, AML patients were divided into 3 clusters based on 29 genes associated with m^7^G and aimed to screen between the 3 clusters for m^7^G-associated candidate genes with potential predictive value. However, deviating from our starting point, we could not find any genes associated with any initial 29 genes as signature genes during the screening process. This may be related to how m^7^G-related regulators mainly achieve biological functions by affecting the methylation levels of substrates. Even so, the clustering based on m^7^G-related genes has given us new hints on tumor purity and immune scoring, based on which we developed a prognostic assessment model featuring 6 genes. A validated line graph including prognostic features and clinical factors was also developed, and correlations between risk profiles and tumor immunity and immunotherapy were also analyzed.

The prognostic scoring model involves 6 characteristic genes, of which high expression of CBR1, CCDC102A, LGALS1, and SLC29A2 are risk factors; high expression of RD3L and TWIST1 are protective factors. Among all these genes, TWIST1 is a vital transcription factor mediating the progression of epithelial-mesenchymal transition and tumor metastasis. Overexpression of TWIST1 can improve the prognosis of AML patients by affecting the cell cycle and enhancing sensitivity to chemotherapeutic agents (19). LGALS1 is mainly involved in the induction of a tolerance program, prompting immune evasion of tumor cells (20). Current studies suggest that LGALS1 exerts tumor-promoting effects by blocking tumor suppressors such as p53 and promoting drug resistance in AML (21, 22). CBR1 single gene polymorphism is significantly associated with Ara-C chemotherapy toxicity (23). CBR1 overexpression can also protect leukemic cells from As O_23_ by regulating reactive oxygen species production while inhibiting CBR1 expression enhances the effect of As O_23_ on therapeutic sensitivity in a variety of leukemia cell lines (24). SLC29A2 primarily encodes the energy non-dependent equilibrium nucleoside transporter protein (ENT2), which transports a wide range of purine and pyrimidine nucleosides (25). In contradiction to our study, TMK-1 cells, a gastric cancer cell line expressing hENT2, were significantly more chemosensitive to Ara-C (26). CCDC102A and RD3L currently lack studies related to AML. CCDC102A belongs to the coiled-coil domain-containing (CCDC) gene. CCDC has many essential biological functions and is thought to be involved in biological behaviors such as proliferation, invasion, and metastasis of malignant tumor cells (27–30). In summary, half of the genes characterized in this model have not been able to fully clarify their functions and mechanisms in AML in the available studies. Therefore, we need more studies to clarify the roles and mechanisms of genes such as CCDC102A, RD3L, and SLC29A2 in AML, which can provide a more reliable theoretical basis for prognostic assessment as screening new therapeutic targets for AML treatment.

In this study, 6 genetic characteristics could predict AML patients’ prognosis compared with traditional AML risk categories (age, etc.). According to the risk score, AML patients could be divided into high-risk and low-risk groups, and a significant difference in survival between the two risk groups were found. In the ROC analysis, the AUC values of 1-year, 3-year, and 5-year survival rates for AML patients in the TCGA cohort were more outstanding than 0.8, indicating a more substantial predictive power than previous studies (3-year AUC of 0.706 and 0.711, respectively). Moreover, after univariate and multivariate analysis, the risk score was identified as an independent prognostic factor for AML. Among all clinical factors, the risk score had the most significant effect on the survival of AML patients and could effectively guide prognostic prediction. Ultimately, a comprehensive prognostic column line graph was constructed combining risk characteristics with clinical parameters. Compared with earlier studies (31), our prognostic assessment system has a better predictive effect with wider applicability. Compared to a recent column-line graph prognostic scoring system based on 18 characterized genes (32), because WGCNA was used to screen subclasses for clusters of characterized genes, our scoring system achieved good predictive results with fewer characterized genes. However, the results verified by qPCR showed that the expression of 6 key genes in AML patients was not different from that of healthy donor, which may be related to the small sample size included, but it does not indicate that it is not related to disease prognosis. From the validation results, there are individual differences in these genes in AML patients, with high expression in some patients and low expression in others. Therefore, it is still necessary to expand the sample size and extend follow-up time for AML to verify the relationship between the expression of these key genes and disease prognosis.

Currently, evasion of antitumor immune response is considered a fundamental cause of AML progression or relapse (33), therefore, immunotherapy is widely studied in the clinical treatment of AML. In the immune evasion mechanism, multiple immune cells are involved. For example, AML plasma may block the effector functions of T and NK cells, increase immunosuppressive cells such as macrophages M2 and monocytes, and decrease immunoreactive cells such as naive B cells and resting mast cells (34). In our study, LGALS1, one of the scoring model signature genes, was closely related to immune infiltrating cells. As a critical regulator of tumor immune evasion, high LGALS1 expression in AML patients is associated with higher macrophage M2 monocyte infiltration (35). Our study also showed a significant correlation between risk score and immune cell infiltration. The risk score was negatively correlated with mast cells, activated CD4^+^ T cells, and positively correlated with 26 other TIL subpopulations. Activated CD4^+^ T cells possess specific effector functions and play an essential role in tumor immunity (36). Resting mast cells are immunoreactive and associated with better survival (37). Thus, the negative correlation of risk score with activated CD4^+^ T cells and mast cells and the positive correlation with other immune cells, suggesting that the 6-characteristic genetic risk score model is closely related to the immune activation status in the tumor microenvironment. Moreover, these findings require more in-depth studies in combination with clinical samples to elucidate the specific relationship between risk scores and the immune microenvironment in AML.

Genetic mutations are another critical cause of tumorigenesis and drug resistance. In our study, we found that KRAS mutations were higher in high-risk patients compared to low-risk patients. Previous studies have demonstrated that clonal mutations in KRAS are associated with treatment resistance (38). Therefore, poor survival in high-risk patients may also be associated with KRAS mutations, which may lead to chemoresistance. Also, high-risk patients have a higher frequency of mutations in genes such as RUNX1, TP53, and ASXL1, which are molecular genetic markers of high risk for AML, which confirms the prognostic reliability of the risk score assessment.

Finally, this study examines the validity of risk scores in predicting response to immunotherapy. Analysis of bladder cancer immunotherapy data showed that patients with prognostic scores distinguished as high risk had lower treatment response rates than low-risk patients. These results suggest that the model can predict immunotherapy response to some extent.

## 5 Conclusion

In this study, a new prognostic scoring model with CBR1, CCDC102A, LGALS1, SLC29A2, RD3L and TWIST1 as the feature genes was developed to predict the prognosis of patients with AML based on their characteristic set of genes for m7G-related clustering. And, there was a significant correlation between this model and tumor immunity. These findings suggested that this scoring model and key risk genes could be used as potential prognostic biomarkers for AML patients.

## Data availability statement

The original contributions presented in the study are included in the article/**Supplementary Material**. Further inquiries can be directed to the corresponding authors.

## Ethics statement

Ethical approval was not required for the study involving humans in accordance with the local legislation and institutional requirements. Written informed consent to participate in this study was not required from the participants or the participants’ legal guardians/next of kin in accordance with the national legislation and the institutional requirements.

## Author contributions

CZ: Methodology, Software, Writing – original draft. RW: Formal Analysis, Funding acquisition, Methodology, Writing – original draft. GW: Data curation, Methodology, Writing – original draft. GL: Formal Analysis, Methodology, Writing – original draft. XW: Formal Analysis, Methodology, Writing – original draft. YG: Data curation, Methodology, Writing – review & editing. ZY: Funding acquisition, Investigation, Project administration, Supervision, Writing – review & editing.

## Supplementary material

Supplementary Figure 1 Distribution of related genes and mutations. **(A)** distribution of m^7^G-related genes on chromosomes; **(B)** Gene mutations.

Supplementary Figure 2 GEO and TARGET external data validation results. **(A)** Risk score, survival time, and survival analysis of GEO external data; **(B)** KM survival curve distribution of GEO external data; **(C)** ROC curve with AUC of GEO external data. **(D)** Risk score, survival time, and survival analysis of TARGET external data; **(E)** KM survival curve distribution of TARGET external data; **(F)** ROC curve with AUC of TARGET external data.

Supplementary Figure 3 GEO external data validation results. **(A)** Risk score, survival time, and survival analysis of GEO external data; **(B)** KM survival curve distribution of GEO external data; **(C)** ROC curve with AUC of GEO external data.

Supplementary Figure 4 TARGET external data validation results. **(A)** Risk score, survival time, and survival analysis of TARGET external data; **(B)** KM survival curve distribution of TARGET external data; **(C)** ROC curve with AUC of TARGET external data.

Supplementary Figure 5 Correlation analysis of clinical characteristics. **(A)** Prognostic KM curves in young samples (Age ≤ 55) and old samples (Age > 55); **(B)** Prognostic KM curves in female and male samples; **(C)** Prognostic KM curves in samples of White or other races; **(D)** Prognostic KM curves in FAB M0~M2 samples or M3~M7 samples; **(E)** Prognosis KM curves in samples with adjuvant treatment.

Supplementary Figure 6 Correlation analysis between risk scores and clinical characteristics and pathological typing. **(A)** Correlation analysis between risk scores and age; **(B)** Correlation analysis between risk scores and gender; **(C)** Correlation analysis between risk scores and race; **(D)** Correlation analysis between risk scores and adjuvant therapy; **(E)** Correlation analysis between risk scores and FAB typing.

Supplementary Figure 7 Correlation between risk model and different immune infiltrating cell types.

Supplementary Figure 8 Immunotherapy Response in prognostic models. **(A)** KM curves for high- and low-risk groups; **(B)** Proportion of patients with different drug responses in the high- and low-risk groups. Cr, complete response; pr, partial response; sd, stable.

Supplementary Figure 9 The mRNA expression of six key risk genes in healthy donors (normal, n=10) and AML patients (n=10).
