# Supplementary figures and images for: Identification and validation of a prognostic risk-scoring model for AML based on m7G-associated gene clustering
Source: Front Oncol. 2024 Jan 11;13:1301236. doi: 10.3389/fonc.2023.1301236 (PMC10808397; doi:10.3389/fonc.2023.1301236)

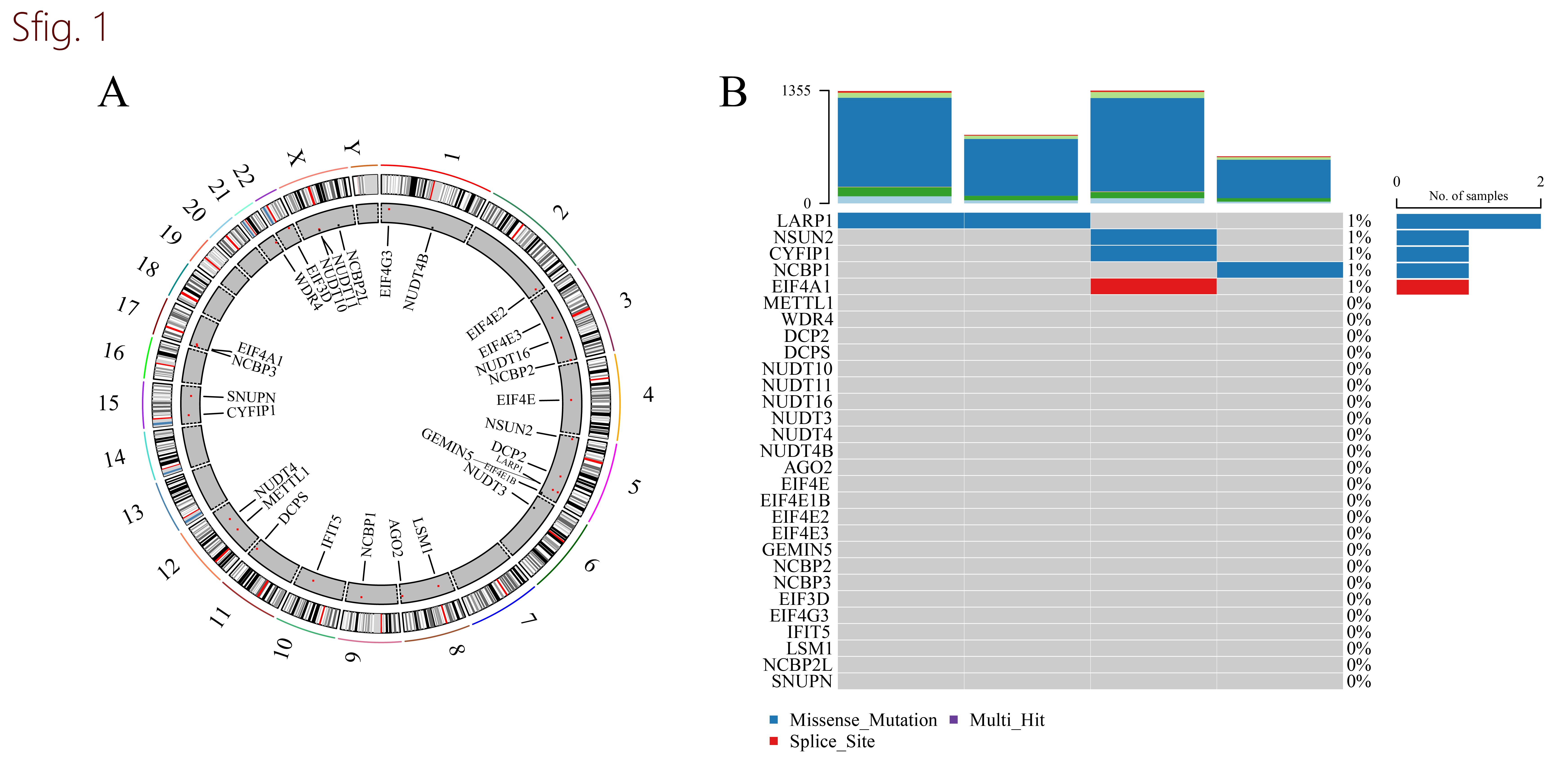

Supplement: Supplementary Figure 1 — Distribution of related genes and mutations. (A) distribution of m7G-related genes on chromosomes; (B) Gene mutations. [file Image_1.tif]

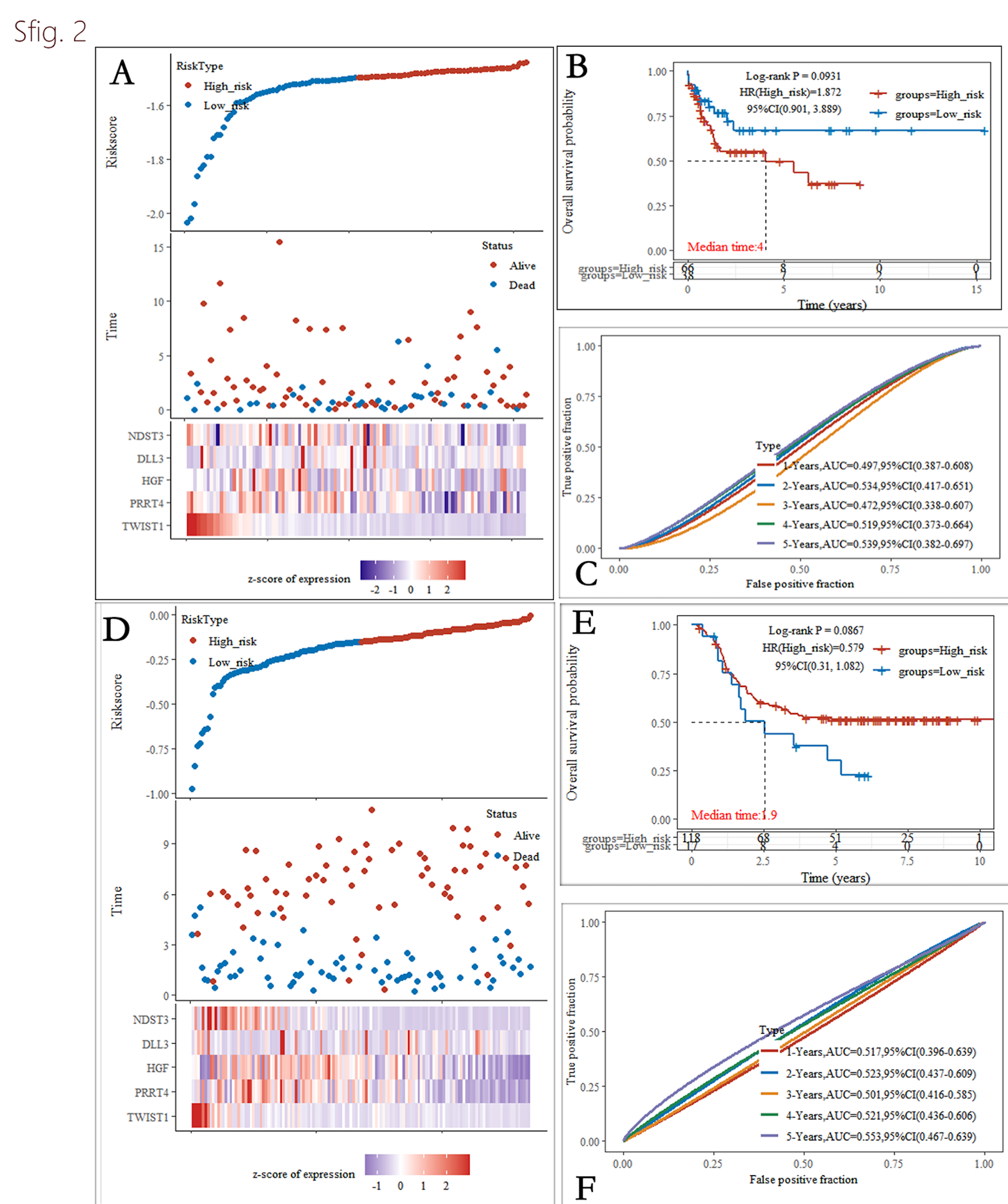

Supplement: Supplementary Figure 2 — GEO and TARGET external data validation results. (A) Risk score, survival time, and survival analysis of GEO external data; (B) KM survival curve distribution of GEO external data; (C) ROC curve with AUC of GEO external data. (D) Risk score, survival time, and survival analysis of TARGET external data; (E) KM survival curve distribution of TARGET external data; (F) ROC curve with AUC of TARGET external data. [file Image_2.tif]

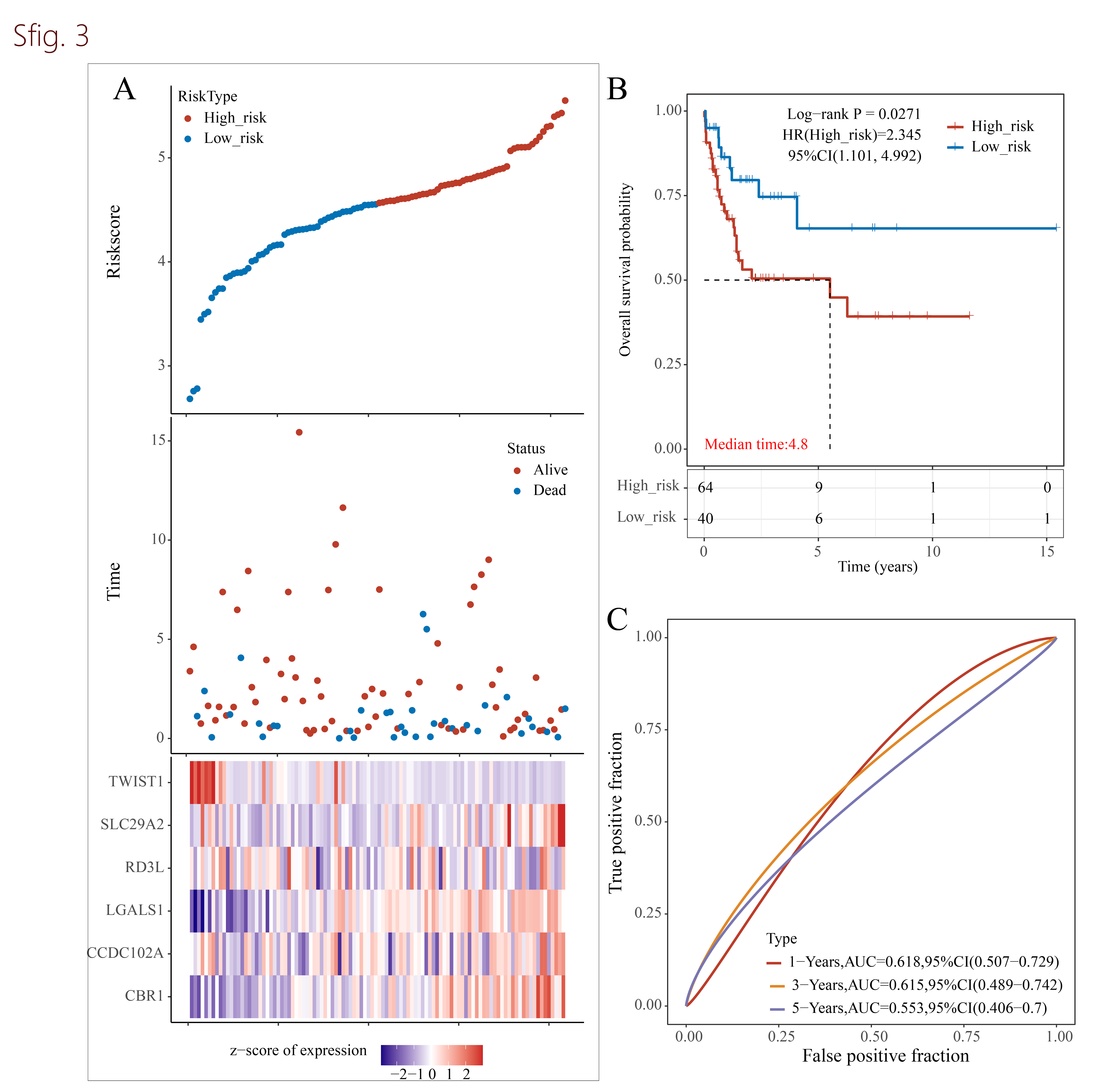

Supplement: Supplementary Figure 3 — GEO external data validation results. (A) Risk score, survival time, and survival analysis of GEO external data; (B) KM survival curve distribution of GEO external data; (C) ROC curve with AUC of GEO external data. [file Image_3.tif]

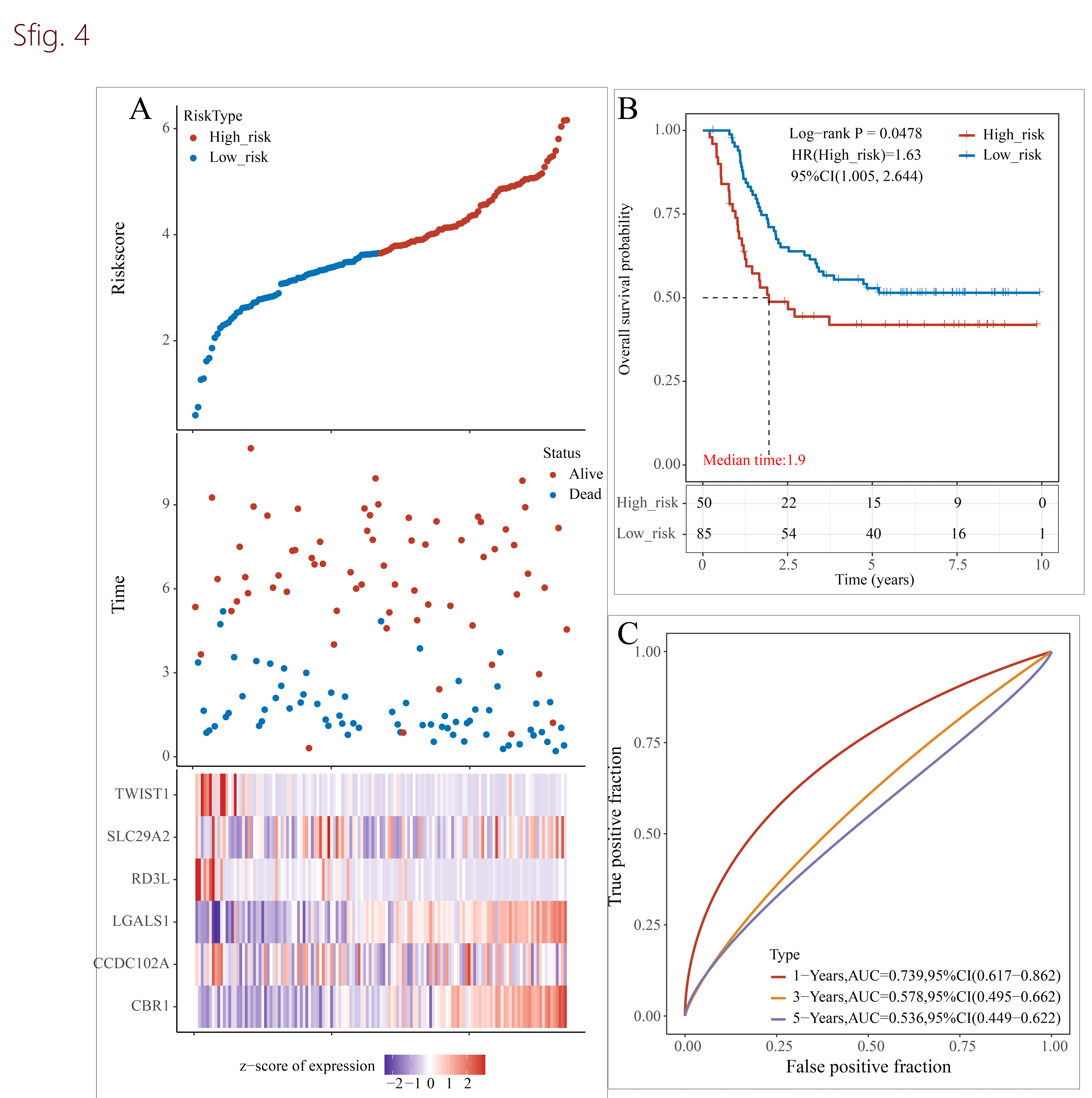

Supplement: Supplementary Figure 4 — TARGET external data validation results. (A) Risk score, survival time, and survival analysis of TARGET external data; (B) KM survival curve distribution of TARGET external data; (C) ROC curve with AUC of TARGET external data. [file Image_4.tif]

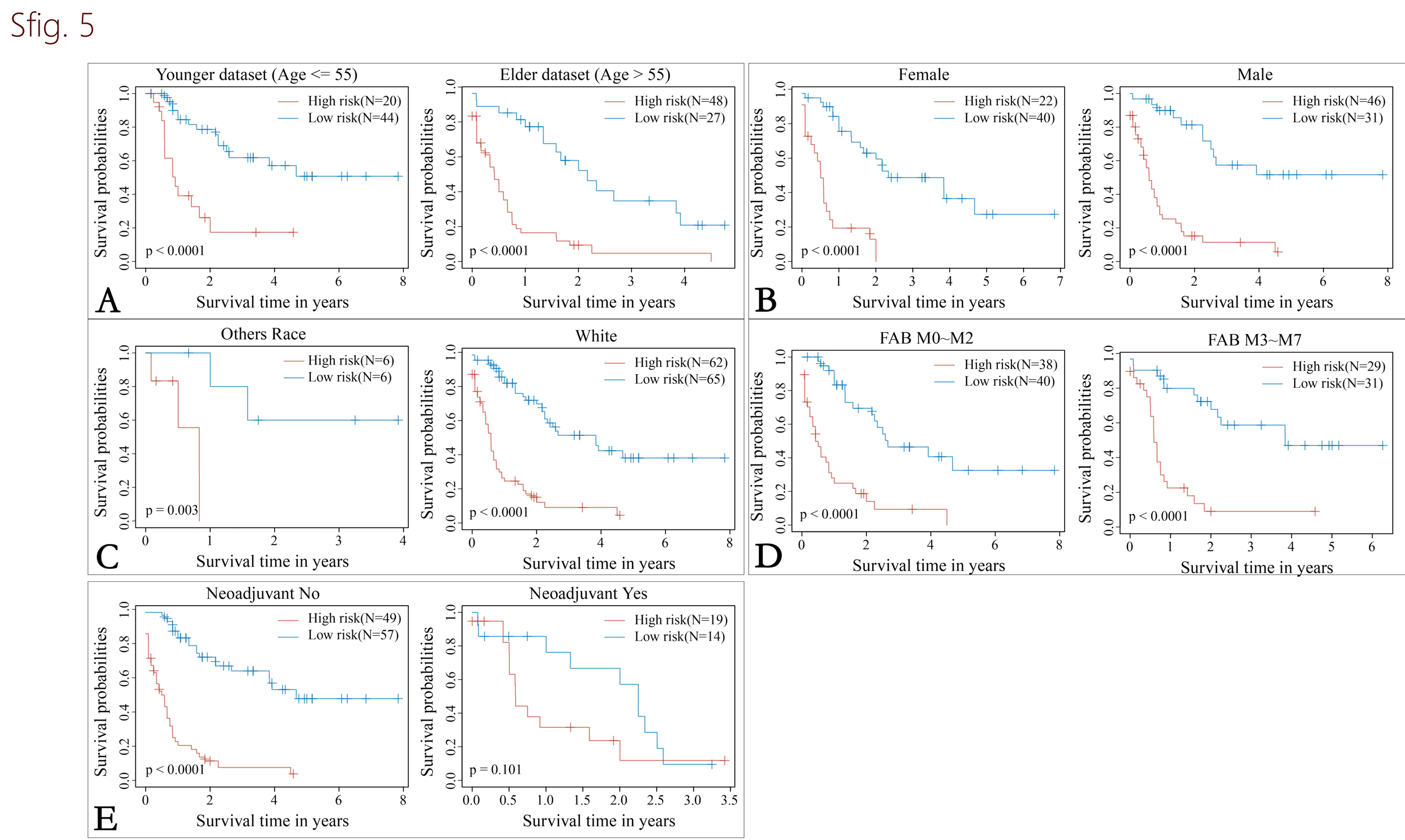

Supplement: Supplementary Figure 5 — Correlation analysis of clinical characteristics. (A) Prognostic KM curves in young samples (Age ≤ 55) and old samples (Age > 55); (B) Prognostic KM curves in female and male samples; (C) Prognostic KM curves in samples of White or other races; (D) Prognostic KM curves in FAB M0~M2 samples or M3~M7 samples; (E) Prognosis KM curves in samples with adjuvant treatment. [file Image_5.tif]

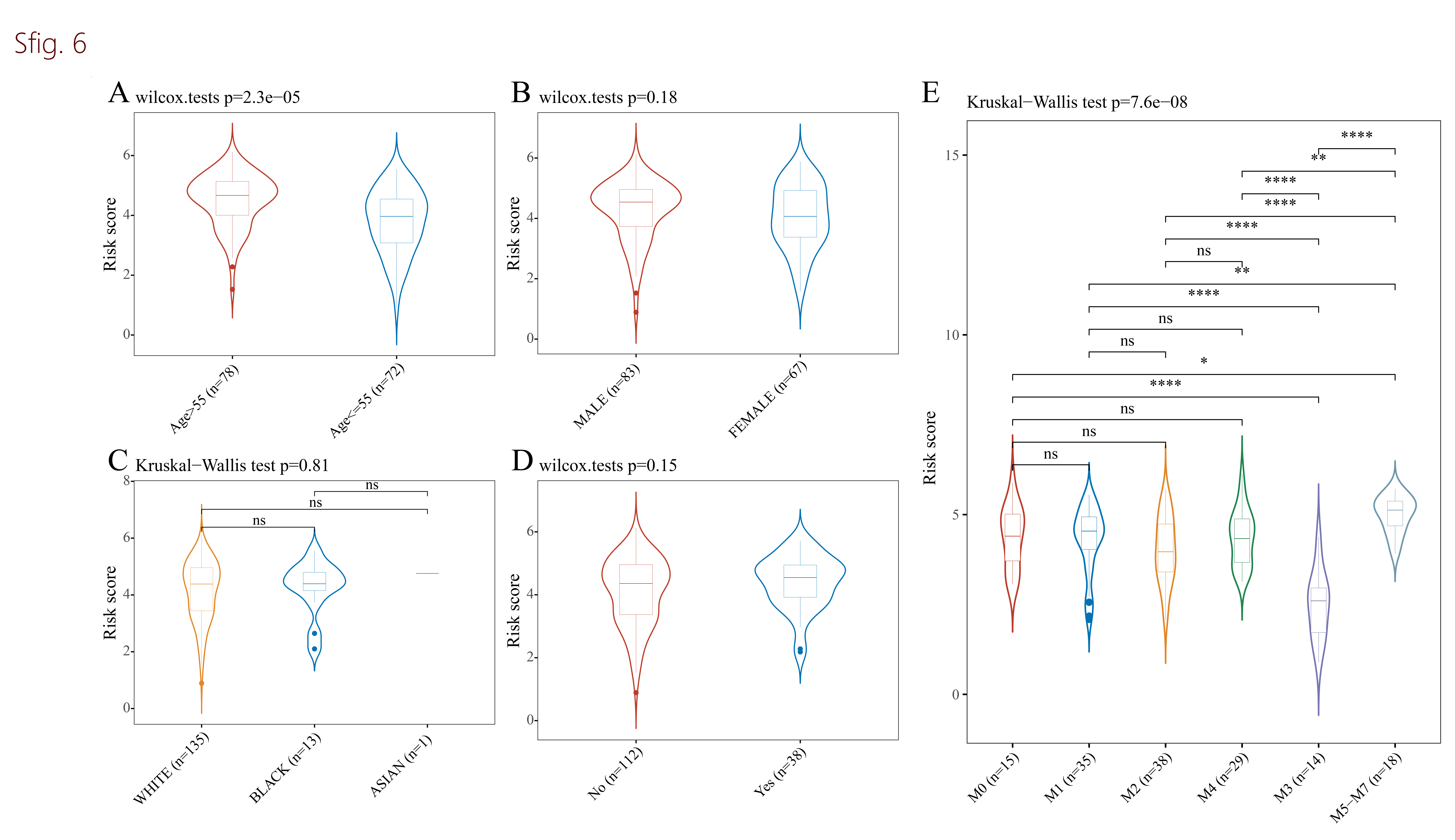

Supplement: Supplementary Figure 6 — Correlation analysis between risk scores and clinical characteristics and pathological typing. (A) Correlation analysis between risk scores and age; (B) Correlation analysis between risk scores and gender; (C) Correlation analysis between risk scores and race; (D) Correlation analysis between risk scores and adjuvant therapy; (E) Correlation analysis between risk scores and FAB typing. [file Image_6.tif]

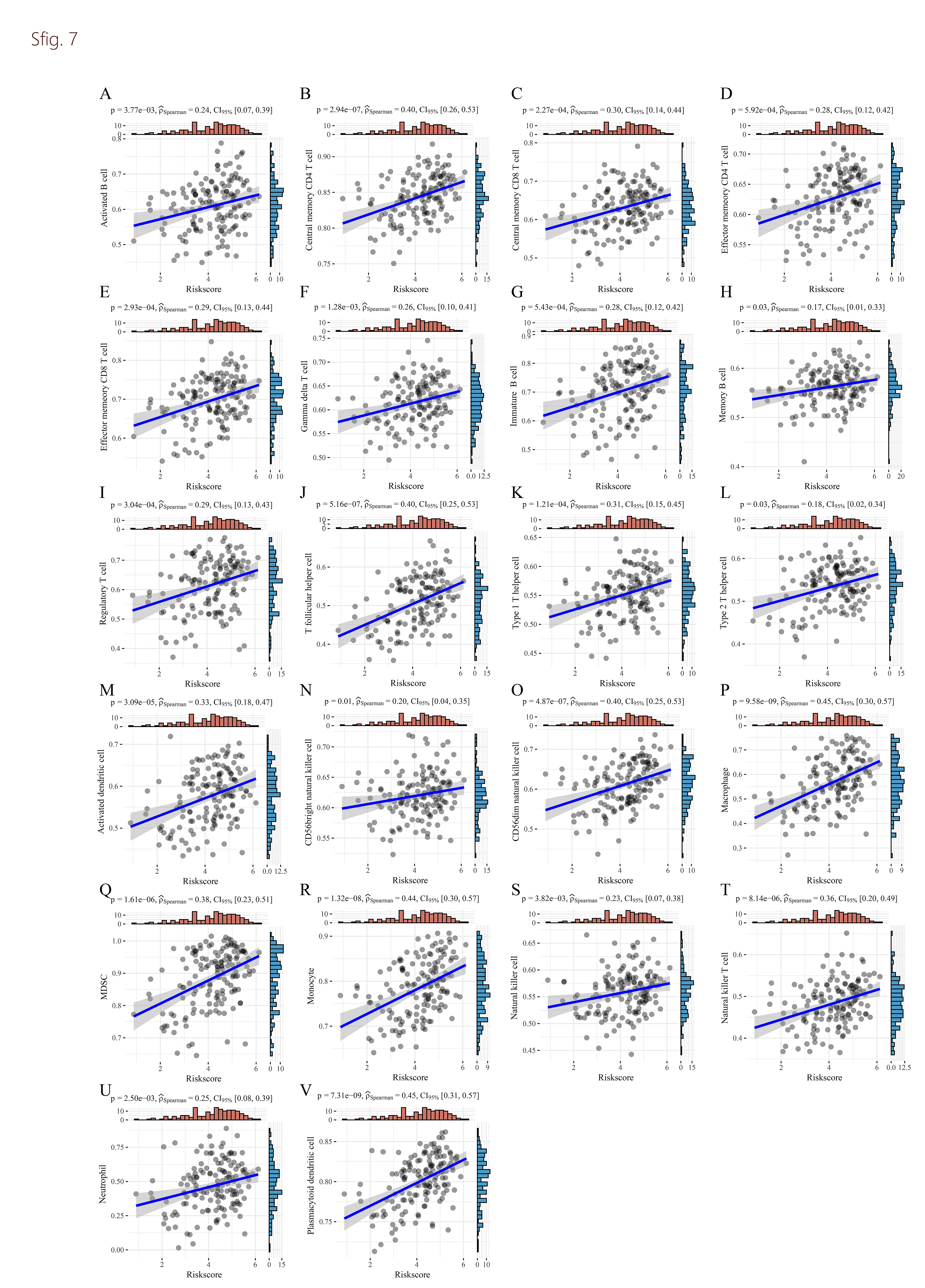

Supplement: Supplementary Figure 7 — Correlation between risk model and different immune infiltrating cell types. [file Image_7.tif]

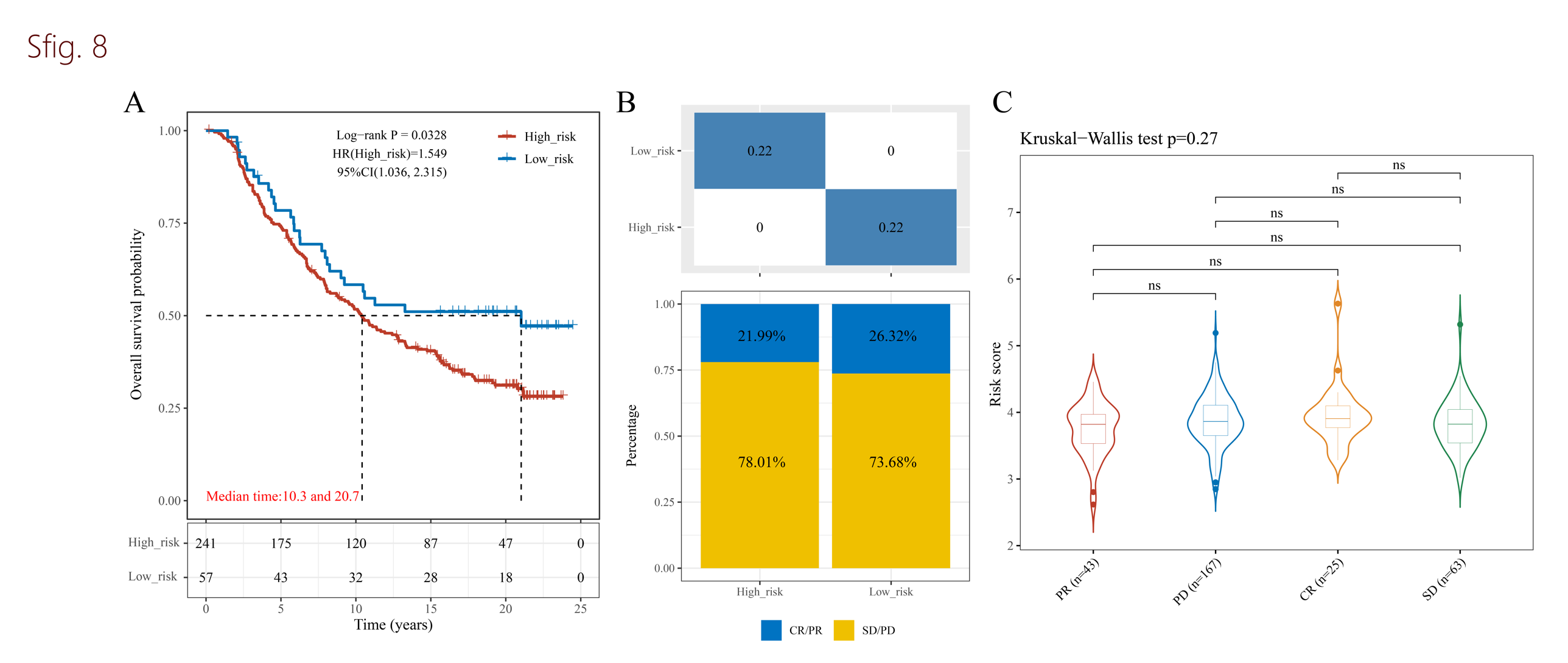

Supplement: Supplementary Figure 8 — Immunotherapy Response in prognostic models. (A) KM curves for high- and low-risk groups; (B) Proportion of patients with different drug responses in the high- and low-risk groups. Cr, complete response; pr, partial response; sd, stable. [file Image_8.tif]

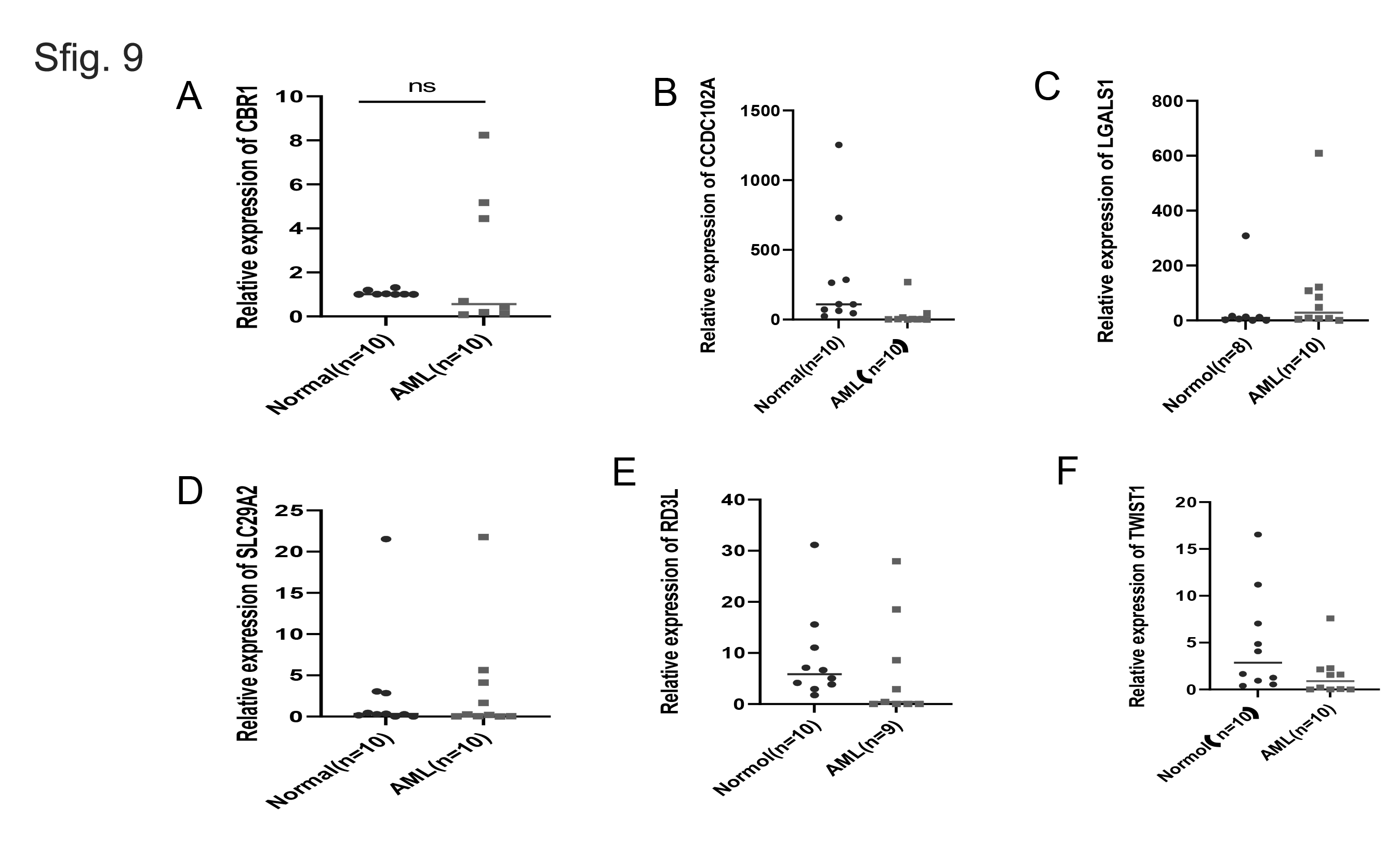

Supplement: Supplementary Figure 9 — The mRNA expression of six key risk genes in healthy donors (normal, n=10) and AML patients (n=10). [file Image_9.tif]
